# Editorial: Immunosuppressive disease in poultry

**DOI:** 10.3389/fimmu.2023.1215513

**Published:** 2023-06-12

**Authors:** Charles Li, Leyi Wang, Shijun Zheng

**Affiliations:** ^1^ Animal Bioscience and Biotechnology Laboratory, Beltsville Agricultural Research Center, Agricultural Research Service, United States Department of Agriculture, Beltsville, MD, United States; ^2^ Veterinary Diagnostic Laboratory, Department of Veterinary Clinical Medicine, College of Veterinary Medicine, University of Illinois, Urbana, IL, United States; ^3^ College of Veterinary Medicine, China Agricultural University, Beijing, China

**Keywords:** immunosuppressive diseases, immunoevasion, poultry, virus, *Eimeria*, major histocompatibility complex

Immunosuppression, originally defined as “a state of temporary or permanent dysfunction of the immune response resulting from damage to the immune system and leading to increased susceptibility to disease” (1), includes suboptimal responses in antibody production, innate and cellular immunities. Immunosuppressive diseases can increase susceptibility to infections and mortalities, reduce feed conversion and vaccine effectiveness, and influence condemnation at processing and total production cost. As a result, they have substantial negative impacts on poultry health and welfare, and production performance in the global poultry industry. Understanding of the pathogenesis of immunosuppressive diseases is crucial to safeguarding health and productivity in the poultry industry.

This Research Topic aims to collect contributions on progresses made in understanding avian immunosuppressive diseases, especially virus-mediated immunosuppression and immunoevasion, persistent infection, pathogen-host interactions, innate immune response, signal transduction, cytokine expressions, and the roles of microRNAs in host antidefense response. It also aims to highlight challenges and opportunities for future research in novel vaccine development, diagnosis, and control of avian immunosuppressive diseases.

With advanced technologies in gene sequencing and the availability of avian immunoreagents, scientists may have more tools in deciphering the pathogenesis of immunosuppressive inducers such as infectious pathogens, and environmental and nutritional/social stressors. This Research Topic keeps readers up-to-date with new insights and advanced knowledge on hosts, interactions between hosts and immunosuppressive pathogens, mechanisms of immunoevasion, and vaccine development in the prevention and control of infectious diseases.

We received eight submissions, and four were published in this Research Topic, including one review focused on the avian leucosis virus-host interaction, one review on the advances of innate immunoevasion by avian immunosuppressive viruses, one original research on the effect of host major histocompatibility complex on pathogenesis and tumorigenesis, and another original research on parasite vaccines in promotion of host responses. This editorial summary highlights these four recent articles published in Frontiers in Immunology that shed light on the mechanisms of immunosuppression in poultry and potential avenues for the prevention and treatment of infectious diseases.

Viruses, including Infectious Bursal Disease Virus (IBDV), Chicken Infectious Anemia Virus (CIAV), Marek’s Disease Virus (MDV) and Avian Leukosis Virus (ALV), are the major immunosuppressive inducers. They can cause apoptosis and/or necrosis of lymphoid cells and induce the malfunction of immune response regulation (2). Among them, ALV is known to initiate diseases correlated with tumor formation and decreased fertility, and induces severe immunosuppression by increasing host vulnerability to other microbial infections and the risk of failure in subsequent vaccination against other diseases. ALV is difficult to control because there is currently no effective vaccine available, and control measures typically involve culling infected birds and strict biosecurity measures to prevent the spread of the virus. Tang et al. reviewed the involvement of host factors in the important molecular events during ALV infection. They revealed the cellular receptors associated with ALV viral entry and displayed how the host’s innate and adaptive immune responses are involved during ALV infection. The authors presented that cellular factors, such as non-coding RNAs and cellular proteins/signaling pathways, were associated with viral replication. They discussed the future perspectives in the development of effective antiviral strategies in chickens.

Innate immunity is the host’s first line of defense against invading pathogens, but some pathogens, particularly immunosuppressive viruses, have evolved sophisticated mechanisms to evade the host’s innate immune responses and survive within the host. Wang et al. reviewed avian immunosuppressive viruses and their evolutionary strategies for evading the innate immune system. The authors identified several viral proteins that interacted with host cellular proteins or regulate microRNA expression to interfere with various components of the innate immune system, such as Toll-like receptors, IFN-I signal transduction, autophagy, apoptosis, necrosis, inflammasome, and metabolic pathways. The review emphasized the importance of understanding of these complex evasion or suppression mechanisms to develop effective antiviral strategies for chickens.

Major histocompatibility complex (MHC)-encoded class I and class II molecules are crucial for mounting specific and optimal adaptive immune responses, particularly in developing vaccinal immunity, and are typically highly polymorphic and polygenic (3). Bertzbach et al. investigated the role of diverse MHC haplotypes in MDV pathogenesis and tumorigenesis in chickens. MDV is a herpesvirus that causes lymphoid hyperplasia and lymphoma, leading to early cytolytic infection of B cells and transformation of T cells, and eventually immunosuppression in infected chickens. The authors found that chickens with certain MHC haplotypes were more genetically susceptible to MDV infection and tumorigenesis than others. The article suggests that breeding for specific MHC haplotypes could decrease the incidence of MDV in poultry flocks.

Coccidian species are a subclass of obligate intracellular protozoan parasites belonging to the apicomplexan class, among which two groups, *Cryptosporidium baileyi* and *Eimeria species*, have been linked to immuno-suppression, although with limited evidence (2). *Eimeria* spp. can cause coccidiosis, a major enteric infectious disease in broiler chickens in the US, resulting in significant economic losses of approximately $12 billion annually in the global poultry industry. Coccidiosis is also a predisposing factor for necrotic enteritis. Chen et al. described an EmARM-β antigen from *Eimeria maxima*, and found that vaccination with recombinant EmARM-β antigen or a plasmid carrying this gene effectively stimulated the expression of Th1 cytokines (IL-2 and IFN-γ), promoted the proportion of CD4^+^ and CD8^+^ T cells and the level of antigen-specific IgG antibodies in immunized chickens, and conferred moderate protective efficacy against *E. maxima* (alleviated weight loss and enteric lesion, reduced oocyst output, and higher anticoccidial index in challenged birds). The authors suggested that this antigen could be a promising candidate for developing a new vaccine against coccidiosis.

Finally, we extend our heartfelt appreciation to the authors who generously shared their original work with us for this Research Topic. We are also grateful to the reviewers who provided us with their insightful comments and helped enhance the quality of the submissions. Our sincere thanks go to the editorial office of Frontiers in Immunology for their exceptional support throughout the process, enabling us to successfully host this Research Topic.

## Author contributions

CL and SZ drafted the manuscript. LW revised it. All authors approved the final version for publication.
